# Clinical Epidemiology and Paraclinical Findings in Tuberculosis Patients in North of Iran

**DOI:** 10.1155/2015/381572

**Published:** 2015-01-28

**Authors:** Farhang Babamahmoodi, Ahmad Alikhani, Jamshid Yazdani Charati, Amir Ghovvati, Fatemeh Ahangarkani, Leila Delavarian, Abdolreza Babamahmoodi

**Affiliations:** ^1^Antimicrobial Resistance Research Center, Department of Infectious Diseases, Mazandaran University of Medical Sciences, Sari, Iran; ^2^Department of Biostatistics, Health Sciences Research Center, Faculty of Health, Mazandaran University of Medical Sciences, Sari, Iran; ^3^Health Management Research Center, Baqiyatallah University of Medical Sciences, Tehran, Iran

## Abstract

*Background*. *Mycobacterium tuberculosis* (*M.TB*) causes a wide spectrum of clinical diseases. The prevalence of TB is different in various parts of Iran and throughout the world. The present study aimed to determine the clinical epidemiology and paraclinical findings of TB. *Methods*. A cross-sectional study was conducted from 2008 to 2013. Patient demographic, clinical, and radiologic characteristics, picked up from the TB patient's files, were collected using a standard questionnaire format. Data was entered and analyzed using the SPSS version 16 statistical software and *P* value < 0.05 was considered statistically significant. *Results*. Out of 212 patients enrolled in this study 62% were male and the mean age was about 50 years old. 98.6% were Iranian, and 46.2% were rural. Prevalence of smear-positive TB was 66.4%. Prevalence of positive PPD was 50.7% with no significant difference between HIV-positive and -negative patients (*P* = 0.8). Prevalence of diabetes mellitus was 17%. 36% of the patients had history of smoking and about 29.3% were addicted to narcotics. Cough was the most common symptom (94.5%) and 84% had sputum. 15 cases (7%) had extrapulmonary TB. The mean time between the onset of symptoms and admission was 46.5 days. The delay for admission between urban and rural populations was not significantly different (*P* = 0.68); but for those who were in prison, the delay was significant (*P* = 0.02). About 46% of the patients had cavitary lesions in CXRs. *Conclusion*. Timely diagnosis of TB especially in prisoners by understanding its most important epidemiologic characteristics and clinical features can help to make an early treatment and prevent spread of mycobacteria and their complications.

## 1. Introduction

Tuberculosis is one of the oldest infectious diseases caused by the bacillus* Mycobacterium tuberculosis* (*M. TB*). TB is typically presented with pulmonary involvement but can also be seen in other organs (extrapulmonary). It is estimated that 70% of HIV-negative smear-positive TB patients die if not treated within 10 years [1].

Tuberculosis still remains a major global health problem. In 2012, an estimated 8.6 million people developed TB with 1.3 million mortalities (including 320,000 deaths among HIV-positive people). TB is more common in men than in women and occurs more often in adults who are in the age of productivity. Of 8.6 million new cases of TB in 2012, about 2.9 million were women and 530,000 were children under 15. The majority of reported TB cases to WHO are from Southeast Asia (29%), Africa (27%), western pacific (19%), India (26%), and China (12%). Of all new cases 1.1 million (13%) were HIV-positive, 75% were from Africa, and 450,000 cases were MDR-TB. [1]. Tuberculosis is associated with risk factors such as age, male gender, HIV infection, smoking, asthma, and family history of close contact with TB patients [2]. The poverty, war, immigration, social disorders, and homelessness play an important role in the spread of tuberculosis [3–5]. Many studies done in other countries have showed that progression of infection to active disease is more rapid among women in childbearing age (25 to 34 y/o) and men above 40 y/o. Smear-positive tuberculosis patients in Iran are reported more commonly among women [6]. Delay in diagnosis and treatment of smear-positive pulmonary tuberculosis leads to the spread of this disease [7]. Clinical manifestations of pulmonary tuberculosis are cough, fever, weakness, and weight loss [8]. According to WHO reports in 2006, this delay in Iran, from the appearance of the first symptoms to treatment, is 127 days. Most of this time is spent on diagnosis processing [9]. Early diagnosis of pulmonary tuberculosis plays a significant role in preventing the spread of the disease and eventual death of the patients [10]. Pulmonary tuberculosis represents a variety of radiographic patterns. These abnormalities depend on age, underlying diseases, and their socioeconomic conditions. Early diagnosis of the disease plays an important role in preventing its irreversible damage and outcomes [11]. Tuberculosis and AIDS have a synergistic effect on each other. The risk of progression of TB, from becoming infected to the presentation of symptoms in HIV-positive cases, is 5% annually and 30% during their life, while these statistics are 5–10% in HIV-negative cases [12, 13]. The prevalence of HIV in tuberculosis patients is 2.2% and mortality rate among HIV-positive cases with tuberculosis is less than 1% [14, 15]. In tuberculosis patients many hematologic findings may be seen which could be normal or abnormal but the most common findings are normocytic, normochromic anemia. Therefore, regarding importance of tuberculosis, especially in a developing country with a large number of Afghan immigrants, growing population, and changes in its age pyramid, it is necessary to have precise information about the disease, such as the outbreak rate, drug resistance, its underlying factors such as diabetes and smoking, social and culture-related factors, and its mortality rate in order to produce appropriate strategies to control the disease and reach World Health Organization's goals. Increasing rate of drug resistance and the prevalence of its risk factors and also its economic, social, and health-related effects, rapid approach in diagnosis, and appropriate treatment will prevent the spread of* Mycobacterium* and its complications. This strategy will be capable of attaining the WHO goal which is controlling the TB at the end of 2015. In this paper, we have studied the clinical epidemiology, laboratory characteristics, and risk factors in tuberculosis inpatients in the north of Iran from 2008 to 2013.

## 2. Materials and Methods

This is a retrospective, descriptive study and routine data analysis was performed. From all the patients admitted to therapeutic centers of infectious diseases in the north of Iran, those who were diagnosed to have tuberculosis (TB) were selected for the study. The place of the study was therapeutic centers of infectious diseases in northern Iran. A total of 212 patients were sampled. Information was taken from the files of the patients with positive or negative smears and extrapulmonary tuberculosis from September 2008 to September 2013. In our study cases were selected according to WHO criteria such as clinical findings, sputum staining, and culture. A questionnaire was used, which was designed based on the goals of the project, and contained demographic, clinical, and radiologic information. The data were entered in the Master Chart and were analyzed using SPSS software version 16 and also phi, chi-square, and *t*-tests. The statistical methods included descriptive and comparative statistical tests to determine the relation between high risk regions in the province (according to epidemiologic studies) and the disease-related data.

## 3. Results

**Figure 1:**
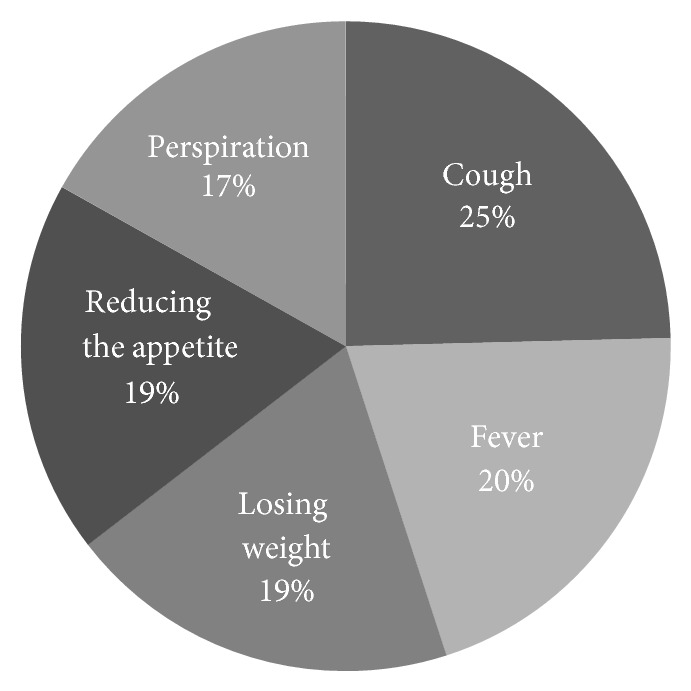
Prevalence of the symptoms.

Out of 212 cases, 131 (62%) were male and 81 (38%) were female. 81.2% were married. The females' age average was 50 and the males' 51 (Table 1). 98.6% of them were Iranian and 1.4% were Afghan. 53.8% of them were urban and 46.2% rural residents. 17% and 10.3% had diabetes and heart failure, respectively. History of smoking was recorded in 36% of the cases and approximately 20% had oral addiction, 6l% was IDUs, and 3.3% were both. Cough was the most common symptom (94.5%) that was productive in 84%. The other symptoms were fever (78.4%), weight loss (75.1%), decreased appetite (71.4%), and sweating (64.8%) (Figure 1).

**Figure 2:**
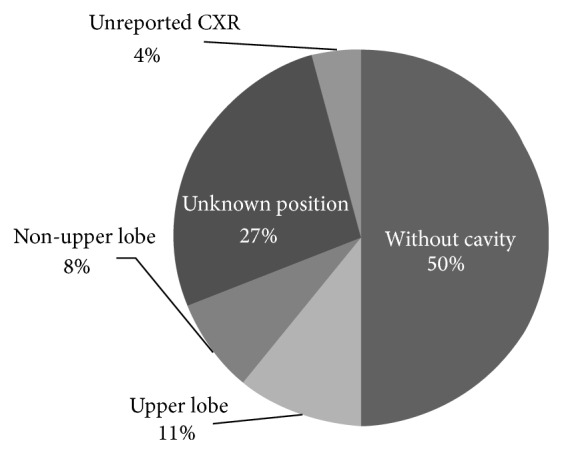
Distribution of the lung cavities in patients.

15 (7%) cases were extrapulmonary tuberculosis: 9 (60%) were male and 6 (40%) were female. Five cases had pleural involvement, 3 cases had bones involvement, 3 had lymphadenitis, 3 had gastrointestinal involvement, and 1 case had concomitant pleura and pericardium involvement. Tuberculosis was seen in the past history of one of the relatives of the patients (14.6% of the cases) and 24% of those had history of treated tuberculosis. We found that 50% of the patients had history of exposure to TB patients. Among 29% of the patients who were tested, HIV was detected in 8% of the cases (27% of total tested patients). 95.7% of the patients had chest radiography report in which 45.7% had cavity and 10.6% of these cavities were located in the upper lobes, 8.4% were in the lower lobes, and 26.7% were in other zones (Figure 2).

**Figure 3:**
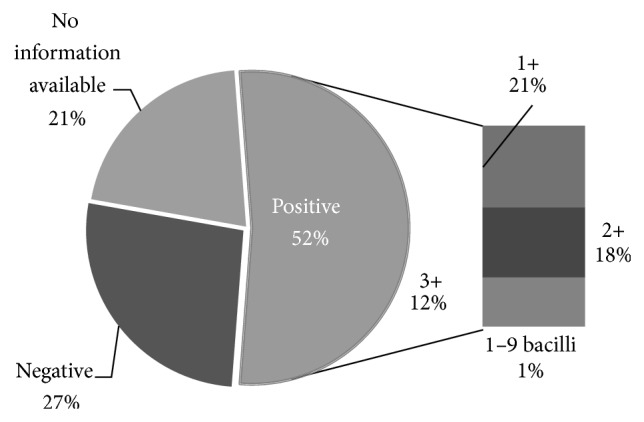
Sputum study before treatment.

On average, prevalence of smear-negative tuberculosis was 26.6% and 52.6% of patients were Smear positive (included 21% of patients 1+, 18% of patients 2+, 12% of patients 3+ and 1% of patients 1–9 bacillus) and there was no available information in 21% of the cases; that is, totally, 33.6% of the patients had negative sputum smear and 66.4% had positive sputum smear (Figure 3).

Among 57% of the cases in which smear was stained in months 2 to 3, 49% were negative, 4.7% were 1+, 3.3% of them were reported to have 1–9 bacilli in their sputum smear, and only one case had positive culture. There was only 1 positive smear in the 3rd to 4th months of follow-up (0.5%). Half of the sputum study was available in the 4th to 5th and last month of follow-up and no positive cases were reported. 69% of the enrolled patients were new cases and the relapse rate was 1%.

Furthermore, 4 cases failed their treatment (1.9%), 4 cases were diagnosed incorrectly, and the mortality rate was 5.7%. Of all cases referred to this center 85% were from another hospital, 8% from our clinic in hospital, 3% from prisons, and about 4% from private clinics. The average time between the first presenting symptom and referring to a doctor was 46.5 days; the average time between medical examination and diagnosis was one day and the average time between diagnosis and starting the treatment was 1 day too (Table 2).

## 4. Discussion and Conclusion

In our study, the incidence of TB in males and females was 62% and 38%, respectively. That is, it was observed 1.6 times as much in males. This difference may be due to more presence of men in the community and more encounters with carriers and the disease's risk factors such as cigarettes and narcotic substances. In the World Health Organization's report in 2013, the ratio of male to female was 1.1 in Iran [1]. In Bandar-Abbas (a southern city in Iran), pulmonary tuberculosis in men is 2.5 times more than in women. In Kashan (a central city in Iran), 50.3% of the disease is seen among women; in Yazd, 53%; in Shiraz, 63%; in Zabol, 64% among men; and in Abadan 55% of cases have been seen among men as well [16]. The last 3 cities are located in the south of Iran. The number of urban patients was more than that of rural ones in our study; the majority of other studies have pointed out this fact as well [17]. It can be due to immigration of villagers to cities and more crowding there and consequently the possibility of prevalence and easy spread of the disease in cities. According to our study, the place of residence (urban or rural) played no role whatsoever in delay between the beginning of symptoms and time of referring to a doctor (*P* = 0.68) which can suggest that there is appropriate availability of medical facilities and clinical centers for villagers just like citizens and so they pursued their disease signs continually and appropriately. The prevalence of cough was the most common symptom in our study. In another research, cough was seen in 75% of the children and it was the most common symptom as well [18]. According to a research conducted by Baghaei et al., cough was the most common symptom (93%) among tuberculosis patients who did not suffer from diabetes [19]. In another study, the prevalence of cough and sputum was 90% and 87%, respectively (the incidence of sputum was 84% in the current study) [20]. The rate of smoking among tuberculosis patients was 36% in our study, which was similar to the finding of another study (34%) by Safa et al. [21]. In this study, average hemoglobin level was 10.1 g/dL and this number for more than 80% of the patients was less than 12 g/dL, which is indicative of considerable anemia. Eishi et al. showed that average hemoglobin level of 85 patients is 12.1 g/dL [22] and in Yekani et al.'s study it was 11.9 g/dL in some patients who were below 65 years old.

In our study, the average age was 50.9 ± 21.19 and the disease was most prevalent among patients who were over 70, which is similar to many other studies [17].

In the present study, the ratio of positive PPD test to all patients who were tested was 50.7%, which is similar to the findings of another research (50%) by Hadadi et al. [23]. However, no significant value was found between PPD and HIV (*P* = 0.08) and a significant relation was seen between cavity of CXR (chest X-ray) and positive PPD (*P* = 0.028).

The ratio of positive smear was 52.4% out of total of 79% available documents in our study; this means that about 65% of smeared patients were positive. According to another study in Golestan (a northern city), 62.7% of pulmonary tuberculosis cases had positive smear [24]. No relation was found between the sputum smear 1 (first time) that was taken before the treatment and PPD (*P* = 0.52), but smears 2 and 3 (second and third time) had a significant relation with PPD (*P* = 0.015, 0.031) before the treatment. On the basis of our findings, 60% of cases with positive PPD had positive smear as well, but 38% of positive smear patients had positive PPD. This clarifies the low sensitivity of PPD compared to smear in our study.

About 80% of the patients had elevated ESR (erythrocyte sedimentation rate) in this study, and it was similar to some other studies [25, 26]. No significant relationship was found between the level of ESR and cavity (*P* = 0.11) and also the average of WBC was 8800/microl, which was similar to another research that was 9100/microl [23].

More than half of the patients in our study had been in contact with a tuberculosis patient. According to a research conducted by Alisjahbana in Indonesia, 52% of their patients were in contact with another patient beforehand [16].

45.7% of our patients had cavities in CXR. However the cavities shown in CXR had no significant relation to the patients' age (*P* = 0.19) and addiction (*P* = 0.6) but those cavities had a strong and direct relation to smoking (*P* = 0.017). Ghasemian et al. found a strong relation between smoking and acquiring pulmonary tuberculosis in 100 patients in Qaemshahr and Sari (northern cities) in 2005. It was also related to the amount of cigarette smoking [27].

85% of the patients were directly referred from the health system network. This clarifies the importance of paying more attention to such centers from technical and medical points of view.

In our study, the delay time from the onset of the symptoms to diagnosis was 46.5 days. In another study in Saudi Arabia, this time was 60 days [28]. According to the World Health Organization's reports, the time from the beginning of the symptoms to the onset of treatment in Iran was 127 days, so delay in diagnosis has been the most time-consuming part [9]. Our findings showed that there is a significant relation to delay between the referring time to a doctor, diagnosis, and treatment in prisons; compared to ordinary people (*P* = 0.02), the weakness in diagnosis and referring system in prisons can be the reason for such problem. In our study, the time required for diagnosis in nonprisoner patients was one day and the average time for starting the treatment was again 1 day. This clarifies that tuberculosis had been diagnosed for most patients before hospitalization.

Considering the nature of tuberculosis as a contagious disease, it is vital to educate people, especially those who are at the risk of getting the disease like family members of TB sufferers, clinicomedical staff, and oldsters, on the ways of transfer of this disease, its prevention, diagnosis, and so forth. It is also required to perform such educational programs continually and regularly every year in order to clarify its importance in various periods of time for people who are exposed to its danger and also ordinary people. Examining and evaluating the conditions needed for incidence and outbreak of tuberculosis in different towns and cities all over the country is also recommended in order to identify high risk regions and perform necessary precautionary measures severely and precisely.

## Figures and Tables

**Table 1:** Age distribution on the basis of gender.

	Age average	Standard deviation	Min.	Max.
Male	51.14	20.6	20	91
Female	50.57	22	15	91

**Table 2:** Delay time at the beginning of symptoms, going to a doctor, and diagnosis (the hospitalization date has been considered the reference time).

Time	Cases	Average	Standard deviation	Min.	Max.
Beginning of the symptoms until seeing a doctor	212	46.5	47	−1	−240
From seeing a doctor until diagnosis	153	−1.71	18.3	−120	31
From diagnosis to treatment	153	−0.64	17.9	−120	31
